# Unveiling the Enigma: Exploring the Intricate Link Between Coronary Microvascular Dysfunction and Takotsubo Cardiomyopathy

**DOI:** 10.7759/cureus.44552

**Published:** 2023-09-01

**Authors:** Pragyamita Datta, Sayandeep Nath, Aniket G Pathade, Seema Yelne

**Affiliations:** 1 Pathology, Jawaharlal Nehru Medical College, Datta Meghe Institute of Higher Education and Research, Wardha, IND; 2 Pathology, Tripura medical college, Agartala, IND; 3 Research and Development, Jawaharlal Nehru Medical College, Datta Meghe Institute of Higher Education and Research, Wardha, IND; 4 Nursing, Shalinitai Meghe College of Nursing, Datta Meghe Institute of Higher Education and Research, Wardha, IND

**Keywords:** cardiovascular medicine, microvascular dysfunction, interplay, management, diagnosis, pathophysiology, tcm, takotsubo cardiomyopathy, cmd, coronary microvascular dysfunction

## Abstract

This review article delves into the intricate and evolving relationship between coronary microvascular dysfunction (CMD) and takotsubo cardiomyopathy (TCM), two intriguing cardiovascular conditions increasingly recognised for their potential interplay. We examine their characteristics, shared pathophysiological mechanisms, diagnostic challenges, and management strategies. Emerging evidence suggests a link between microvascular dysfunction and the development of TCM, leading to a deeper exploration of their connection. Accurate diagnosis of both conditions becomes essential, as microvascular dysfunction may modify TCM outcomes. We underscore the significance of understanding this connection for improved patient care, emphasising the need for tailored interventions when CMD and TCM coexist. Collaborative research and heightened clinical awareness are advocated to advance our comprehension of this relationship. Through interdisciplinary efforts, we aim to refine diagnostic precision, develop targeted therapies, and enhance patient outcomes in cardiovascular medicine.

## Introduction and background

Medical interest has surged toward two distinct yet captivating cardiovascular conditions: coronary microvascular dysfunction (CMD) and takotsubo cardiomyopathy (TCM). While initially perceived as separate entities, recent research hints at a potentially intricate link between these conditions. This article delves into this complex interplay between CMD and TCM, probing their shared pathophysiological mechanisms, clinical presentations, diagnostic challenges, and treatment implications [1-3].

Coronary microvascular dysfunction encompasses a spectrum of disorders characterised by anomalies in the microvasculature's structure and function within the coronary circulation. Unlike traditional coronary artery disease, which affects large epicardial vessels, CMD involves dysfunction in the smaller arterioles and capillaries that regulate myocardial blood flow. Despite the absence of significant obstructive coronary artery disease, CMD leads to angina-like symptoms, myocardial ischemia, and abnormal stress test results. The elusive nature of CMD and its limited diagnostic tools pose challenges in diagnosis, rendering understanding its underlying mechanisms and clinical significance increasingly pivotal [4,5].

In contrast, TCM, or "broken heart syndrome," manifests as transient left ventricular dysfunction mimicking acute myocardial infarction. Chest pain, electrocardiographic changes, and elevated cardiac biomarkers typify this condition. Often arising from emotional or physical stressors, TCM's hallmark is the unique apical ballooning pattern evident in cardiac imaging. While initially viewed as reversible with a favourable prognosis, recent research illuminates potential severe complications and enduring consequences [6,7].

The realisation of a possible connection between CMD and TCM carries profound ramifications for both clinical practice and research. Evidence suggests that patients with TCM often exhibit microvascular dysfunction, hinting at an underlying link between these conditions. Understanding this association could offer insights into the triggers and pathogenesis of TCM. Furthermore, discerning the relationship between CMD and TCM stands to shape therapeutic strategies and enhance patient outcomes [8,9].

This review comprehensively explores the evolving relationship between CMD and TCM. We offer a holistic perspective on their shared pathophysiological mechanisms, clinical manifestations, diagnostic hurdles, and therapeutic implications by delving into the crossroads of these two distinct yet potentially intertwined conditions. Through a critical analysis of existing research, clinical observations, and theoretical models, our synthesis aims to provide healthcare professionals, researchers, and clinicians with an exhaustive resource. This resource illuminates conceivable connections between CMD and TCM, emphasising the importance of recognising and addressing this association in clinical practice. Our in-depth examination of CMD and TCM's complexities strives to advance medical understanding, enhance patient care, and formulate precise strategies for managing these captivating cardiovascular conditions.

## Review

Coronary microvascular dysfunction (CMD)

Definition and Pathophysiology of CMD

Coronary microvascular dysfunction encompasses a spectrum of disorders characterised by intricate abnormalities within the smaller coronary vessels, including arterioles and capillaries. In contrast to traditional coronary artery disease, which primarily affects the larger epicardial vessels, CMD homes in on the delicate microcirculation. To comprehensively understand CMD's pathophysiology, it's crucial to explore the underlying mechanisms that have been identified. Endothelial dysfunction stands at the forefront of CMD's pathophysiology, playing a pivotal role in impairing the functionality of the microvasculature. This dysfunction is intricately linked to the imbalance of vasoactive factors and nitric oxide availability, ultimately affecting the vascular tone and endothelium-dependent vasodilation [10]. Moreover, vascular remodelling, a dynamic process involving vessel structure and composition changes, contributes significantly to CMD. The maladaptive remodelling of arterioles and capillaries alters their responsiveness and regulatory capacity, affecting myocardial blood flow control. Intriguingly, the autonomic nervous system's influence on microvascular function cannot be overlooked. Altered autonomic regulation, marked by a disruption in the balance between sympathetic and parasympathetic activity, further exacerbates CMD. This imbalance can trigger abnormal vasoconstriction and vasodilation responses, contributing to inadequate myocardial perfusion. We can draw insights from recent research to delve deeper into CMD's multifaceted pathophysiology [10].

Clinical Presentation and Diagnostic Challenges

The clinical presentation of CMD closely mirrors that of obstructive coronary artery disease, making it challenging to distinguish between the two based solely on symptoms. Patients with CMD often report chest pain, dyspnea (shortness of breath), and exercise intolerance, commonly associated with traditional coronary artery disease. This similarity in symptoms can lead to confusion in diagnosis, mainly when relying solely on clinical presentation [11].

One of the critical diagnostic challenges in CMD lies in the limitations of conventional diagnostic tests, such as angiography. Unlike obstructive coronary artery disease, CMD does not involve significant narrowing or stenosis of the larger coronary arteries, typically visualised through angiography. This absence of visually apparent coronary stenosis can lead to underdiagnosis or misdiagnosis, as these tests may not capture the microvascular abnormalities characteristic of CMD [12].

Furthermore, the need for well-defined diagnostic criteria and standardised assessments for CMD exacerbates the difficulty in identifying the condition accurately. The absence of universally accepted guidelines to diagnose CMD makes it challenging for healthcare practitioners to consistently recognise and differentiate it from other cardiac conditions. This lack of standardised criteria also hampers research efforts and makes it difficult to compare findings across studies [13].

The confluence of overlapping symptoms, along with the limited sensitivity of existing diagnostic tests, contributes to a significant underrecognition and underdiagnosis of CMD. This diagnostic shortfall is consequential, potentially resulting in treatment delays and impacting patient outcomes. An appreciable aspect to consider is the percentage of undetected cases due to these challenges. Additionally, it is pertinent to discuss the sensitivity and specificity of the employed diagnostic tests and the symptoms associated with CMD. As the landscape of CMD research evolves, the need becomes apparent to establish standardised diagnostic criteria and innovative diagnostic techniques. By addressing this pressing need, the accurate identification and effective management of this intricate condition can be significantly improved [14].

Underlying Causes and Risk Factors

Coronary microvascular dysfunction is a complex condition influenced by various underlying causes and risk factors extending beyond traditional cardiovascular risk factors. While hypertension, diabetes, and dyslipidemia are recognised as conventional contributors to CMD, a growing body of evidence highlights the significance of non-traditional risk factors in its development [15].

Psychosocial stress emerges as a potent contributor to CMD. Chronic stress triggers the release of stress hormones, leading to endothelial dysfunction and impaired vasomotor responses in the coronary microvasculature. This can result in reduced myocardial blood flow regulation and contribute to the manifestation of CMD symptoms [16].

Hormonal imbalances play a role in CMD pathogenesis, particularly in women. Oestrogen, for instance, exerts protective effects on endothelial function, and its decline during menopause might contribute to microvascular dysfunction. Hormonal fluctuations during the menstrual cycle also influence vasomotor responses, potentially impacting coronary microcirculation [17].

Systemic inflammation is increasingly recognised as a critical player in CMD. Chronic inflammation contributes to endothelial dysfunction, oxidative stress, and impaired nitric oxide bioavailability. Inflammatory cytokines can directly affect coronary microvascular function, altering vasodilation responses and contributing to CMD [18].

Endothelial dysfunction, a hallmark feature of CMD, is a common link between these risk factors. It disrupts the delicate balance between vasodilation and vasoconstriction, impairing coronary blood flow regulation. Endothelial dysfunction contributes to the microvascular abnormalities observed in CMD and lays the groundwork for developing ischemic symptoms [19,20].

Available Diagnostic Techniques for CMD

Coronary flow reserve measurements using Doppler ultrasound: Doppler ultrasound measures blood flow velocities within the coronary arteries during rest and stress. The coronary flow reserve is assessed by calculating the ratio of flow velocities before and after vasodilation. Reduced coronary flow reserve indicates microvascular dysfunction and impaired vasodilatory capacity, which can contribute to myocardial ischemia [21,22].

Positron emission tomography (PET): Positron emission tomography imaging quantitatively assesses myocardial blood flow at rest and during stress, usually induced by pharmacological agents or exercise. The comparison of these flow measurements provides insights into the functionality of the microvasculature. Positron emission tomography can help identify regions of reduced perfusion and abnormalities in microvascular function, contributing to the diagnosis of CMD [23,24].

Cardiac MRI: Cardiac MRI offers non-invasive visualisation of the myocardium and surrounding structures. Techniques like myocardial perfusion imaging provide information about blood flow distribution, aiding in identifying microvascular dysfunction. Cardiac MRI can help evaluate myocardial oxygen supply-demand balance and detect ischemic regions without obstructive coronary artery disease [25,26].

Invasive coronary reactivity testing: Invasive procedures involving the administration of vasoactive agents within the coronary arteries allow for direct assessment of microvascular function. Endothelial-dependent and independent vasodilatory responses are measured, providing insights into microvascular dysfunction. However, the invasiveness of this approach limits its routine clinical use [27,28].

Despite the availability of these diagnostic techniques, challenges persist in interpreting results and establishing definitive diagnostic criteria. The lack of standardised protocols and reference values makes establishing universally accepted thresholds for diagnosing CMD difficult. Variability in patient populations, equipment, and procedural protocols further complicates result interpretation. Overcoming these challenges requires collaborative efforts among researchers and clinicians to establish consensus guidelines and refine the diagnostic approach for CMD, ultimately enhancing its recognition and management [29].

Current Treatment Strategies for CMD

Lifestyle modifications: Lifestyle interventions stand as the cornerstone of CMD management. Encouraging patients to adopt a heart-healthy lifestyle is paramount. This includes regular physical exercise tailored to individual capabilities, as exercise has been shown to enhance endothelial function and improve microvascular health. Furthermore, weight management plays a pivotal role, as excess body weight can contribute to metabolic disturbances and exacerbate microvascular dysfunction. Smoking cessation is of utmost importance, given that smoking damages endothelial cells and worsens microvascular health. Additionally, addressing psychosocial stressors through stress reduction techniques, mindfulness, and psychological counselling can contribute to overall well-being and potentially mitigate the impact of stress on microvascular function [30,31].

Pharmacological interventions: Pharmacotherapy for CMD aims to alleviate symptoms, enhance microvascular function, and target underlying risk factors. Endothelial function-enhancing agents, such as angiotensin-converting enzyme (ACE) inhibitors and statins, promise to improve endothelial health and promote vasodilation. Vasodilators, such as calcium channel blockers and nitric oxide donors, can help alleviate microvascular constriction and enhance coronary blood flow. Additionally, anti-inflammatory agents are being explored due to their potential role in reducing vascular inflammation and improving microvascular function [32,33].

Patient education and psychosocial support: Patient education is integral to holistic CMD management. Empowering patients with knowledge about their condition, its underlying mechanisms, and the significance of lifestyle modifications fosters active participation in their care. Moreover, addressing psychosocial stressors, including anxiety and depression, is essential, as these factors can exacerbate microvascular dysfunction. Psychological support and counselling can help patients cope with CMD’s emotional and psychological impact, promoting a holistic approach to well-being [34,35].

Takotsubo cardiomyopathy (TCM)

Definition and Characteristic Features of TCM

Takotsubo cardiomyopathy, often called "broken heart syndrome," is a unique and transient condition characterised by acute left ventricular dysfunction, typically presenting with symptoms resembling acute myocardial infarction. The hallmark feature of TCM is the distinctive ventricular ballooning observed during cardiac imaging, which resembles a Japanese octopus trap known as "takotsubo." Left ventricular dysfunction typically involves the apex but can also affect other heart segments [36].

Proposed Mechanisms for TCM Development

The precise mechanisms driving the development of TCM remain a subject of continuous investigation and exploration. Multiple hypotheses have emerged, each offering insights into the intricate cascade of events that culminate in this unique cardiac phenomenon. These mechanisms include but are not limited to, surge-induced myocardial stunning, microvascular dysfunction, coronary artery vasospasm, and neurogenic influences [37].

Catecholamine surge-induced myocardial stunning: One of the prominent theories centres around the significant role of the autonomic nervous system's response to stress. A sudden and excessive release of stress hormones, particularly catecholamines such as adrenaline, is thought to trigger a surge-induced myocardial stunning. This stunning effect results in transient myocardial dysfunction, marked by impaired contractility and ventricular ballooning [38].

Microvascular dysfunction: Another hypothesis emphasises the role of microvascular dysfunction in TCM development. Under the influence of stress hormones, there is potential for the small coronary vessels, including arterioles and capillaries, to undergo dysregulation. Microvascular dysfunction disrupts normal blood flow regulation, leading to inadequate myocardial oxygen supply. This compromised perfusion contributes to myocardial stunning and the characteristic ventricular ballooning observed in TCM cases [39].

Coronary artery vasospasm: The involvement of coronary artery vasospasm is also proposed as a mechanism for TCM initiation. Stress-induced alterations in vasomotor tone could trigger transient spasms in the coronary arteries, leading to reduced blood flow to the heart muscle. This diminished blood supply can result in myocardial dysfunction, with the apical region particularly susceptible due to its anatomical characteristics [40].

Neurogenic influences: Neurogenic factors, including the brain-heart connection, have gained attention as potential contributors to TCM development. Emotional stressors can stimulate the autonomic nervous system, leading to altered heart rate variability, sympathetic nervous system activation, and hormonal fluctuations. This complex interplay between the brain and the heart might contribute to the intricate pathophysiology of TCM [41].

Clinical Presentation and Diagnostic Criteria for TCM

Takotsubo cardiomyopathy is characterised by a distinctive clinical presentation and diagnostic criteria that differentiate it from other cardiovascular conditions. The hallmark symptom is the sudden onset of chest pain, often accompanied by dyspnea, palpitations, and a feeling of impending doom. This presentation can closely mimic acute coronary syndrome, particularly ST-segment elevation myocardial infarction (STEMI), leading to initial diagnostic challenges [42].

Diagnostic criteria for TCM have evolved to aid in accurate differentiation from other cardiac pathologies. Transient left ventricular dysfunction is a central feature of TCM, with echocardiography and cardiac MRI serving as crucial tools for its visualisation. Coronary angiography reveals the absence of significant coronary artery stenosis or plaque rupture, further supporting the diagnosis. This differentiation is paramount, as TCM is managed differently from acute coronary syndromes [43].

Also, diagnostic criteria exclude other conditions that might mimic TCM's presentation. Pheochromocytoma, a rare tumour causing excess catecholamine release, and myocarditis, inflammation of the heart muscle, should be ruled out due to their potential to induce similar stress-related myocardial dysfunction [44].

Cardiac imaging, particularly echocardiography and cardiac MRI is indispensable in confirming the diagnosis. Echocardiography unveils the hallmark feature of TCM, the unique apical ballooning pattern, which distinguishes it from other cardiac pathologies. Cardiac MRI further enhances diagnostic accuracy by providing detailed insights into myocardial tissue characteristics and perfusion patterns, facilitating the differentiation of TCM from other conditions presenting with similar symptoms [45].

Differentiating TCM from Acute Coronary Syndrome

Distinguishing TCM from acute coronary syndrome (ACS) is paramount due to their shared clinical presentations, which often include chest pain, dyspnea, and electrocardiographic changes. One crucial distinction is the absence of culprit coronary lesions on angiography in TCM cases. Unlike ACS, where obstructive coronary artery disease is typically evident, TCM patients display normal or near-normal coronary arteries. This disparity emphasises the need for comprehensive angiographic evaluation to exclude coronary artery obstruction and confirm the absence of significant stenosis [46].

Furthermore, TCM patients tend to exhibit less pronounced elevations in cardiac biomarkers, such as troponin levels, when compared to the more typical ACS cases. While troponin elevation is a hallmark of myocardial injury, the extent of elevation in TCM is often milder than observed in ACS, further aiding differentiation. However, it is essential to note that a certain degree of troponin elevation can still be present in TCM cases, underscoring the importance of integrating multiple diagnostic criteria [47].

Cardiac imaging is an invaluable tool in the differentiation process, particularly echocardiography and cardiac MRI. The characteristic apical ballooning pattern observed during imaging is a hallmark of TCM. This distinctive morphology, resembling the shape of a Japanese octopus trap (takotsubo), is a strong differentiator from ACS. Imaging findings directly visualise ventricular dysfunction and shape abnormalities, providing critical insights into the diagnosis [48].

Management and Prognosis of TCM

Takotsubo cardiomyopathy management encompasses a multi-faceted approach focused on supportive care to stabilise hemodynamics, address complications, and alleviate symptoms. Given the acute nature of TCM, patients often require vigilant monitoring in a specialised cardiac care setting. Stabilising hemodynamics involves carefully managing fluid balance, blood pressure, and heart rate to ensure optimal cardiac function. Efforts to maintain adequate perfusion and oxygenation are crucial, and interventions like supplemental oxygen and intravenous medications might be employed as necessary [37].

Furthermore, addressing potential complications is of paramount importance in the management of TCM. Patients with TCM are at risk of developing heart failure due to transient left ventricular dysfunction. Standard heart failure therapies, including diuretics and beta-blockers, may be considered to manage volume overload and control heart rate. Arrhythmias, such as atrial fibrillation or ventricular tachycardia, might occur in the acute phase and necessitate appropriate treatment to restore a stable cardiac rhythm. Additionally, systemic embolism, particularly involving the left ventricular clot or thrombus formation, is a potential complication that requires attention to prevent embolic events [49].

Most TCM cases exhibit a favourable prognosis with the spontaneous recovery of left ventricular function within weeks to months. Serial cardiac imaging, such as echocardiography or cardiac MRI, is valuable for monitoring the improvement in ventricular function over time. The heart's remarkable ability to heal and return to normal functioning underscores the transient nature of TCM. However, vigilance remains essential, as some cases might experience a protracted recovery period or recurrent episodes [50].

Lifestyle modifications and addressing psychosocial factors play a crucial role in the long-term prognosis of TCM patients. Encouraging patients to adopt heart-healthy habits, including maintaining a balanced diet, engaging in regular physical activity, and avoiding tobacco and excessive alcohol consumption, contributes to overall cardiovascular well-being. Addressing underlying stressors and providing psychological support is equally important, as emotional triggers often precede TCM episodes. Comprehensive care extends beyond the acute phase to mitigate the potential triggers for recurrent episodes and optimise long-term cardiac health [51].

The interplay between CMD and TCM

Review of Studies Suggesting a Connection between CMD and TCM

Recent years have witnessed a burgeoning body of research that unveils the captivating prospect of a multifaceted interplay between CMD and TCM. This intriguing association has emerged through meticulously scrutinising diverse studies, including case reports and retrospective analyses. These inquiries have illuminated instances where TCM manifested in individuals previously displaying indications of underlying microvascular dysfunction. These preliminary observations have ignited curiosity within the medical community, suggesting a compelling nexus between CMD and TCM's pathogenesis [52].

Numerous case reports have documented instances in which individuals diagnosed with CMD, an ailment marked by coronary microvasculature irregularities, subsequently encountered episodes of TCM. These cases potentially offer insights into a shared physiological connection that warrants closer investigation. Likewise, retrospective patient data analyses have unveiled a suggestive pattern that a subset of TCM cases might be heralded by documented or suspected CMD, implying a temporal relationship deserving thorough exploration [53].

While these revelations prompt inquiries into potential mechanisms and implications, they also underline the need to consider additional research methodologies. Could microvascular dysfunction contribute to or even initiate TCM's development? Could CMD influence the trajectory or prognosis of TCM episodes? Rooted in these clinically observed correlations, these queries beckon a deeper understanding of the shared pathophysiological underpinnings that might underscore this intriguing association [54].

The studies discussed undoubtedly build a compelling case for the connection between CMD and TCM. Nevertheless, they simultaneously serve as a launchpad for broader research endeavours. The implications of such an interrelationship could resonate profoundly, impacting diagnostic precision, risk evaluation, and therapeutic strategies. Hence, the clarion call for further investigation into these intricate dynamics transcends academic curiosity. It promises to advance comprehension of these enigmatic cardiovascular conditions and, by extension, elevate patient care in a substantive and impactful manner [55-57].

Shared Pathophysiological Mechanisms

Endothelial dysfunction: A central component in both CMD and TCM, endothelial dysfunction refers to the impaired function of the endothelium, the inner lining of blood vessels. In CMD, compromised endothelial function reduces vasodilation and impairs blood flow regulation within the coronary microvasculature. Similarly, endothelial dysfunction could hinder normal coronary blood flow responses in TCM, potentially contributing to the transient myocardial dysfunction observed during TCM episodes [58].

Oxidative stress: Elevated oxidative stress, characterised by an imbalance between the production of reactive oxygen species (ROS) and the body's antioxidant defences, is a common denominator in CMD and TCM. In CMD, oxidative stress can lead to endothelial dysfunction and structural alterations in the microvasculature, further impairing blood flow regulation. Takotsubo cardiomyopathy episodes, often triggered by stress, can trigger a surge in oxidative stress, which may contribute to myocardial injury and contractile dysfunction [59].

Neurohormonal pathways: Dysregulation of neurohormonal pathways, particularly the autonomic nervous system's response to stress, plays a pivotal role in both conditions. Excessive release of stress hormones, such as adrenaline, can lead to vasoconstriction, increased heart rate, and myocardial oxygen demand. In individuals with pre-existing CMD, this heightened neurohormonal response could trigger the myocardium towards the transient dysfunction observed in TCM [60].

Abnormal microvascular function and autonomic responses: Aberrant microvascular function, a hallmark of CMD, contributes to impaired myocardial perfusion and ischemia. In the context of TCM, microvascular dysfunction could exacerbate the myocardial stunning observed during TCM episodes. Furthermore, abnormal autonomic nervous system responses, including sympathetic overactivity, could influence both conditions, affecting coronary blood flow and myocardial contractility [61].

Potential Role of Microvascular Dysfunction in Triggering TCM

Microvascular dysfunction is a compelling candidate for triggering TCM in specific vulnerable individuals. Coronary microvascular dysfunction, marked by compromised vasodilation and impaired myocardial perfusion in the microcirculation, could set the stage for a cascade of events contributing to the initiation of TCM. The intricate link between these conditions is grounded in the notion that microvascular dysfunction might make the myocardium susceptible to the profound physiological responses of stress-induced catecholamine release [62].

In individuals with CMD, the altered microvascular tone and reduced perfusion capacity might weaken the myocardium's resilience against the surge of stress hormones, particularly adrenaline. Stress-induced catecholamines have been implicated in TCM as potential triggers, initiating a sequence of cardiac events that lead to the distinctive features of this condition. The heightened vulnerability created by microvascular dysfunction could predispose the myocardium to stress-induced myocardial stunning, whereby the heart's contractility becomes impaired and its ability to pump blood effectively diminishes. This state of myocardial dysfunction, combined with the surge of catecholamines, could culminate in the hallmark ventricular ballooning pattern observed in TCM, particularly affecting the apex of the heart [63].

Unravelling the intricate connection between microvascular dysfunction and TCM could provide valuable insights into the complex mechanisms governing the onset of TCM. By understanding how microvascular dysfunction amplifies the effects of stress hormones on the heart, researchers and clinicians can develop a more nuanced understanding of the factors contributing to TCM development. This comprehension, in turn, could inform targeted therapeutic approaches that address microvascular dysfunction and stress hormone responses, potentially mitigating the risk of TCM episodes in susceptible individuals [8].

Impact of CMD on the Prognosis and Outcomes of TCM

Emerging evidence underscores the potential significance of CMD in shaping the clinical course of TCM. Research suggests that pre-existing CMD in individuals experiencing TCM could influence disease severity and subsequent clinical outcomes. This interaction between CMD and TCM holds implications for risk stratification and the development of tailored management strategies [64].

In the context of TCM episodes, individuals with underlying CMD may exhibit more pronounced left ventricular dysfunction. The compromised microvascular function observed in CMD might render the myocardium vulnerable to the stress-induced catecholamine surge that characterises TCM. Consequently, the acute myocardial stunning and contractile impairment associated with TCM might manifest more severely in those with microvascular dysfunction. This could contribute to a heightened likelihood of complications, including heart failure and arrhythmias, impacting the overall prognosis [65].

Recognising CMD as a potential modifier of TCM outcomes is crucial for risk assessment and management decisions. Risk stratification becomes more nuanced when CMD coexists with TCM, as these individuals might warrant closer monitoring and intensive interventions to mitigate the heightened risk of adverse events. Tailored management strategies could involve a more proactive approach to managing cardiac function, optimising fluid balance, and instituting preventative measures against potential complications. Such individualised management aligns with the complex interplay between CMD and TCM, addressing the unique vulnerabilities presented by their coexistence [66].

Incorporating the potential impact of CMD into the risk assessment and management of TCM provides a broader perspective on the factors influencing disease progression and outcomes. As understanding this interaction evolves, healthcare practitioners can better tailor their approach to patient care, ensuring that individuals with CMD and TCM receive a comprehensive evaluation and strategic interventions that optimise their clinical trajectory [67].

Theoretical Models Explaining the Relationship between CMD and TCM

Exploring the intricate relationship between CMD and TCM has given rise to several theoretical models to unravel the underlying mechanisms that potentially connect these conditions. These models provide valuable insights into the complex interplay between CMD and TCM, shedding light on possible pathways that link these distinct cardiovascular entities [68].

The "two-hit hypothesis": This model postulates that CMD is the first "hit," rendering the myocardium more vulnerable to subsequent stressors, such as catecholamine release triggered by emotional or physical stress. In this scenario, microvascular dysfunction could act as a priming factor, altering myocardial responsiveness and making it more susceptible to the detrimental effects of stress hormones. This susceptibility could lead to myocardial stunning and the unique contractile abnormalities observed in TCM. The "two-hit hypothesis" suggests that CMD sets the stage for the development of TCM by modifying the myocardium's response to stressors [69].

The "common substrate hypothesis": According to this model, CMD and TCM share underlying mechanisms that make them mutually predisposing conditions. These shared mechanisms could include abnormalities in neurohormonal signalling, endothelial dysfunction, and impaired autonomic regulation. Factors that contribute to CMD, such as chronic stress or metabolic disturbances, could also create an environment conducive to the development of TCM under the right circumstances. The common substrate hypothesis underscores the potential interconnectedness of CMD and TCM, proposing that they arise from an everyday pathophysiological basis [70]. Exploring these theoretical models enriches our comprehension of the intricate connection between CMD and TCM. While these models provide valuable frameworks for understanding the potential relationship, it's essential to acknowledge that the exact mechanisms driving this connection are still the subject of ongoing research. Through the pursuit of these models and continued investigation, we can move closer to uncovering the intricate web that links these two intriguing cardiovascular conditions.

Clinical assessment and diagnostic challenges

Challenges in Diagnosing CMD and TCM Individually

Diagnosing CMD and TCM as distinct cardiovascular conditions presents significant diagnostic challenges due to their unique complexities [71].

Coronary microvascular dysfunction's subtle presentation and diagnostic complexity: Coronary microvascular dysfunction's inconspicuous and often asymptomatic nature adds to the difficulty of its identification. Unlike typical angina associated with traditional coronary artery disease, CMD often displays vague symptoms or remains entirely asymptomatic. Recognizing CMD becomes particularly challenging as patients may lack characteristic chest pain, leading to delayed diagnosis or overlooking the condition. Moreover, the absence of well-defined diagnostic criteria for CMD further compounds accurate detection [12].

Diverse diagnostic criteria complicate CMD identification: The absence of universally agreed-upon diagnostic criteria for CMD contributes significantly to the diagnostic predicament. Varied definitions and diagnostic approaches across different medical contexts hinder establishing a consistent identification method. This lack of standardized criteria can lead to underdiagnosis or misclassification, impeding the implementation of appropriate management strategies [72].

Takotsubo cardiomyopathy's resemblance to ACS and diagnostic confusion: Takotsubo cardiomyopathy's clinical presentation resembles ACS with symptoms like chest pain, electrocardiographic changes similar to STEMI, and elevated cardiac biomarkers, which can cause diagnostic confusion. The overlap between TCM and ACS can result in healthcare providers selecting ACS treatment strategies without considering the possibility of TCM. Consequently, TCM patients might receive interventions like invasive coronary procedures or antithrombotic therapy that might not suit their condition [73].

Implications of misdiagnosis: Misdiagnosing CMD and TCM can lead to inappropriate treatment plans and interventions. Mistakenly categorizing CMD patients as having non-cardiac causes for their symptoms could delay proper management, potentially exacerbating underlying microvascular dysfunction. Similarly, misidentifying TCM as ACS might expose patients to unnecessary invasive procedures and therapies, subjecting them to undue risks and potential complications [74].

Overlapping Clinical Features and Diagnostic Dilemmas

The convergence of clinical features between CMD and TCM challenges their accurate diagnosis. Patients with both CMD and TCM may present with overlapping symptoms, including angina-like chest pain, breathlessness, and abnormal results in stress tests. The shared clinical presentation of these conditions accentuates the complexity of discerning one from the other. The diagnostic dilemma arises because these symptoms are common in various cardiac disorders, making it arduous to attribute them to CMD or TCM solely based on clinical presentation [75].

In times of diagnostic uncertainty, the importance of employing precise diagnostic tools becomes evident. These tools must go beyond identifying symptoms and delve into each condition's underlying mechanisms and structural abnormalities. While symptoms might be the initial presentation, accurate differentiation necessitates comprehensive imaging techniques and functional assessments that illuminate the distinct pathophysiological underpinnings of CMD and TCM. This not only aids in pinpointing the correct diagnosis but also forms the foundation for developing targeted management strategies [76].

Appropriate management hinges on accurate diagnosis, especially given the potential impact of shared symptoms on patient outcomes. The pursuit of enhanced diagnostic precision has driven the exploration of advanced imaging modalities such as cardiac MRI, which can delineate the myocardial microstructure and function, aiding in differentiating CMD from TCM. Moreover, non-invasive functional assessments like coronary flow reserve measurements using Doppler ultrasound offer insights into microvascular function and help discern between these conditions. The development and validation of these diagnostic tools are pivotal in overcoming the challenges posed by overlapping clinical features and ensuring optimal patient care [77].

Novel Imaging and Diagnostic Techniques to Assess CMD and TCM

The continuous evolution of imaging and diagnostic technologies has paved the way for innovative methods of assessing CMD and TCM. These advancements offer promising avenues to enhance our understanding and diagnostic capabilities for these conditions [52].

Cardiac MRI with contrast-enhanced perfusion imaging: Cardiac MRI has emerged as a powerful tool for evaluating microvascular dysfunction in CMD. Utilising contrast-enhanced perfusion imaging, cardiac MRI allows for a non-invasive assessment of myocardial blood flow and perfusion patterns. This technique helps identify regions of impaired microvascular function and provides valuable insights into the subtler aspects of CMD. Moreover, cardiac MRI's ability to visualise the unique apical ballooning pattern associated with TCM aids in distinguishing it from other conditions with similar clinical presentations [78].

Invasive techniques for microvascular assessment: Invasive techniques, such as coronary reactivity testing and Doppler ultrasound, are increasingly recognised for their potential in assessing microvascular function. Coronary reactivity testing involves administering pharmacological agents to induce controlled vasodilation and measuring the coronary blood flow response. This approach allows for the direct evaluation of microvascular reactivity and provides valuable information about the functional state of the microvasculature. Similarly, Doppler ultrasound offers insights into blood flow velocities within the microcirculation, aiding in identifying abnormalities in blood flow regulation [79].

These novel techniques promise to improve the accuracy and specificity of diagnosing CMD and TCM. By enabling the direct assessment of microvascular function and subtle myocardial changes, these imaging and diagnostic methods enhance our ability to differentiate between these conditions and guide targeted treatment strategies. As these technologies continue to evolve, they are likely to play an increasingly significant role in the clinical management of CMD and TCM, facilitating earlier diagnosis and more personalised patient care [80].

Importance of Accurate Diagnosis for Appropriate Management

Accurate diagnosis is a cornerstone for effectively managing CMD and TCM. The ramifications of misdiagnosis reverberate across patient outcomes, potentially causing profound harm. A misdiagnosed CMD might lead to inappropriate therapeutic interventions that neglect its unique pathophysiology, thereby failing to mitigate underlying microvascular dysfunction. This mismanagement could perpetuate the progression of microvascular impairment, contributing to a cascade of adverse cardiovascular events. Similarly, inaccurately managed TCM could translate into inadequate care for a condition that requires distinct attention. This misalignment in management could impede the natural recovery process and exacerbate the associated symptoms and complications [15].

A comprehensive diagnostic approach is thus paramount to tailoring treatment strategies precisely to the specific condition at hand. Recognising CMD and TCM individually and understanding their potential coexistence inform appropriate therapeutic decisions. Accurate diagnosis empowers healthcare professionals to provide targeted interventions that address the intricate mechanisms driving each condition. Such precision ensures that interventions are aligned with the underlying pathophysiology, optimising the chances of positive patient outcomes. Through an accurate and holistic diagnostic approach, clinicians can circumvent the pitfalls of misdiagnosis, avoid unnecessary interventions, and significantly enhance patient well-being by offering the most fitting and efficacious treatments [81].

Management strategies

Conventional Treatment Approaches for CMD

Managing CMD entails a comprehensive and multifaceted strategy that focuses on addressing the underlying risk factors contributing to microvascular dysfunction and improving the health and function of the microvasculature itself. This approach aims to alleviate symptoms, improve myocardial perfusion, and enhance cardiovascular health [3].

Lifestyle modifications: A cornerstone of CMD management is empowering patients to adopt healthy lifestyle practices. Encouraging individuals to embrace a heart-healthy diet is crucial, emphasising the consumption of nutrient-rich foods such as fruits, vegetables, whole grains, lean proteins, and healthy fats while minimising processed foods, sugary drinks, and excessive sodium intake. This dietary approach supports blood pressure control, lipid management, and weight maintenance, ultimately promoting better microvascular health [82].

Regular exercise: Physical activity improves endothelial function, enhances microvascular dilation, and increases cardiac fitness. Tailored exercise programmes that combine aerobic activities, strength training, and flexibility exercises can improve blood flow regulation and oxygen delivery to the myocardium. Regular exercise also positively impacts systemic factors such as blood pressure, lipid levels, and glucose metabolism, which are crucial in mitigating microvascular dysfunction [83].

Stress reduction: Stress reduction techniques are pivotal in CMD management. Chronic stress can contribute to endothelial dysfunction and exacerbate microvascular abnormalities. Incorporating relaxation techniques such as deep breathing, meditation, and yoga can help mitigate the effects of stress on the cardiovascular system. Stress reduction also involves addressing psychosocial factors and adopting strategies to manage daily stressors effectively [84].

Smoking cessation: Smoking significantly contributes to endothelial dysfunction and microvascular damage. Encouraging individuals to quit smoking is paramount to improving microvascular health and reducing the risk of cardiovascular events. Smoking cessation interventions, including counselling and pharmacotherapy, can significantly enhance endothelial function [85].

Pharmacological interventions: Pharmacotherapy often addresses associated risk factors and enhances microvascular function. Angiotensin-converting enzyme (ACE) inhibitors manage hypertension and improve endothelial function by reducing vascular resistance. Statins are beneficial for managing dyslipidemia and mitigating inflammation, which can contribute to microvascular dysfunction. Antiplatelet agents, such as aspirin, may be prescribed to reduce the risk of thrombotic events and improve microvascular flow [86].

Current Management Strategies for TCM

Monitoring and assessment: Continuous monitoring of hemodynamics, fluid balance, and cardiac biomarkers is paramount to evaluating the extent of ventricular dysfunction, assessing fluid status, and tracking improvements. Regular blood pressure, heart rate, and oxygen saturation assessments help gauge the patient's cardiovascular stability and guide adjustments in medical management [87].

Fluid management: Optimal fluid management is crucial to maintaining an appropriate balance while preventing fluid overload or depletion. Careful monitoring of urine output and central venous pressure aids in tailoring fluid administration, especially in the acute phase. Diuretics may be used judiciously to manage fluid retention and oedema [88].

Complication prevention: Preventing complications is a cornerstone of TCM management. Standard heart failure therapies, including angiotensin-converting enzyme inhibitors, beta-blockers, and diuretics, might be considered in patients with significant ventricular dysfunction. These medications aim to alleviate symptoms, stabilise hemodynamics, and enhance cardiac function [89].

Spontaneous recovery: One of the remarkable aspects of TCM is its tendency towards spontaneous recovery. Most TCM cases exhibit gradual improvement in left ventricular function over time. This recovery is a testament to the transient nature of the condition, although the timeline varies between individuals. Regular echocardiographic assessments are essential to monitor improvements and guide the adjustment of medical therapy [90].

Long-term follow-up and psychological support: Although TCM cases typically show favourable outcomes, long-term follow-up is recommended to ensure sustained recovery and identify any potential recurrence. Psychological support is an integral component of TCM management, as the emotional stress that triggers TCM can leave a lasting impact on patients. Addressing the psychosocial aspects through counselling and support groups can aid emotional healing [91].

Special Considerations When CMD and TCM Coexist

When confronted with cases where both CMD and TCM coexist within an individual, an intricate treatment approach is imperative. The convergence of these conditions necessitates a holistic strategy that navigates the complexities of both CMD and TCM, acknowledging their potential interrelation. Central to this approach is recognising the profound impact that microvascular dysfunction, a hallmark of CMD, can exert on the outcomes of TCM [2].

A critical step in managing such cases is meticulously evaluating individual patient characteristics. This evaluation extends beyond the confines of CMD and TCM, encompassing the patient's broader medical history, comorbidities, and risk factors. These factors play a pivotal role in shaping the optimal treatment path. Comprehensive risk assessment guides the selection of therapies, ensuring that interventions are tailored to address the unique nuances of both conditions while considering potential interactions and complications [92].

The personalised approach in this context acknowledges the intricate interplay between CMD and TCM. Rather than viewing these conditions in isolation, healthcare practitioners should perceive them as interconnected components of the patient's cardiovascular landscape. Tailoring interventions to this interplay enhances patient care, yielding a more effective treatment regimen. This personalised strategy may encompass lifestyle modifications, pharmacological interventions, and psychological support, all seamlessly integrated to address both CMD and TCM harmoniously [93].

By adopting this holistic and personalised treatment paradigm, healthcare professionals optimise patient management in cases where CMD and TCM coexist. This approach reflects a nuanced understanding of their potential relationship, emphasising the need to acknowledge the complex interplay between these conditions for the most effective and comprehensive patient care [94].

Potential Future Therapies Targeting Both CMD and TCM

Improving microvascular function: Emerging research suggests that interventions designed to enhance microvascular function could hold significant promise for both CMD and TCM. Therapies focused on endothelial health and vasodilation might alleviate the microvascular dysfunction common to both conditions, potentially preventing the cascade of events that lead to TCM. By improving coronary blood flow regulation, these interventions could mitigate the susceptibility of CMD patients to TCM episodes triggered by stressors [95].

Mitigating the effects of stress hormones: Given the role of stress hormones, particularly adrenaline, in triggering TCM, future therapies could aim to modulate the effects of these hormones on the cardiovascular system. Developing agents that buffer the impact of stress-induced catecholamines might prevent or attenuate the myocardial stunning characteristic of TCM. These interventions could safeguard individuals with CMD, who might be at higher risk of developing TCM due to underlying microvascular dysfunction [96].

Modulating neurohormonal pathways: Neurohormonal imbalances are implicated in both CMD and TCM, suggesting that therapies targeting these pathways could benefit both conditions. Agents that influence autonomic nervous system responses, regulate sympathetic activation, and modulate the release of stress hormones could provide a comprehensive approach to managing the intricate relationship between CMD and TCM. By restoring balance to these pathways, such interventions improve microvascular function and reduce susceptibility to TCM [97].

Collaborative efforts between cardiology subfields: The convergence of CMD and TCM highlights the importance of collaboration among various cardiology subfields. Cardiologists specialising in microvascular disorders and those focused on TCM could work synergistically to develop targeted interventions. A collaborative approach could involve integrating expertise in vascular health, heart failure management, and neurohormonal regulation, resulting in therapies that effectively address the shared pathophysiological mechanisms underlying CMD and TCM [98].

Future directions and research implications

Areas for Further Research into the CMD-TCM Relationship

The intricate connection between CMD and TCM remains an area for further exploration. Research should delve deeper into understanding the underlying mechanisms that link these conditions, such as the role of shared neurohormonal pathways and their impact on microvascular function. Prospective studies could investigate whether CMD predisposes individuals to TCM and how CMD might modify TCM outcomes. Longitudinal studies assessing the prevalence of TCM in patients with documented CMD could help elucidate the temporal relationship between the two conditions.

Novel Treatment Avenues Based on Understanding the Link

The evolving understanding of the potential interplay between CMD and TCM could lead to novel treatment approaches that simultaneously target both conditions. Strategies focusing on improving microvascular function, addressing endothelial dysfunction, and modulating the impact of stress hormones hold promise in preventing or mitigating TCM episodes in susceptible individuals. Exploring the potential synergies between therapies used for CMD and TCM could yield innovative interventions that improve patient outcomes.

Importance of Collaborative Research Between Cardiology Disciplines

Given the potential overlap between CMD and TCM, collaborative research efforts among various cardiology disciplines are paramount. Cardiologists specialising in microvascular disorders and those focused on TCM should work together to uncover shared mechanisms, define diagnostic criteria, and develop comprehensive management strategies. A multidisciplinary approach integrating expertise from diverse fields, including cardiology, endocrinology, and psychology, can offer a more comprehensive understanding of the complex relationship between these conditions.

## Conclusions

In conclusion, this comprehensive review has illuminated the intricate relationship between CMD and TCM, shedding light on their potential interconnection. Through thoroughly exploring their distinct characteristics, shared pathophysiological mechanisms, diagnostic challenges, and management strategies, we have unveiled the intriguing possibility that microvascular dysfunction may contribute to the development and outcomes of TCM. This understanding has significant implications for patient care, underscoring the importance of accurate diagnosis and tailored interventions, particularly in cases where CMD and TCM coexist. As we navigate the evolving landscape of cardiovascular medicine, we advocate for ongoing collaborative research efforts and heightened clinical awareness to unravel the complexities of this relationship. By doing so, we strive to enhance diagnostic precision, refine therapeutic approaches, and ultimately improve the well-being and outcomes of individuals affected by these fascinating cardiovascular conditions.
